# The Application of “Stilted Building” Technique in the Embolization of Aneurysms with Secondary Branches

**DOI:** 10.1155/2021/9976541

**Published:** 2021-06-20

**Authors:** Yi Qi, Yongquan Sun, Yang Wang, Jianwen Jia, Hongliang Zhong, Hongchao Yang, Ming Lv, He Liu

**Affiliations:** ^1^Department of Neurosurgery, Beijing Chaoyang Hospital, Capital Medical University, Beijing, China; ^2^Department of Interventional Neuroradiology, Tiantan Hospital, Capital Medical University, Beijing, China

## Abstract

**Objective:**

Many intracranial aneurysms often have branch arteries, and it is especially important to protect them during embolization. The purpose of the present study was to evaluate the curative effect and safety of the “stilted building” technique.

**Methods:**

25 patients with intracranial aneurysms with branch arteries that have been treated by coil embolization with the “stilted building” technique were retrospectively reviewed. Clinical follow-up was performed after endovascular treatment.

**Results:**

All 25 patients successfully underwent aneurysm embolization. During the operation, the ruptured sac and most of the body of the aneurysm were embolized using the “stilted building” technique. Immediate imaging showed that the blood flow to the branch arteries from the neck or sidewall of the aneurysm was unobstructed. The mRS scores of the 25 patients during the follow-up period were mRS 0 for twenty-one patients, mRS 1 for three patients, and mRS 6 for one patient. No aneurysms recurred among the patients who completed the follow-up.

**Conclusions:**

In an aneurysm with a branch artery, when a balloon or stent cannot be effectively used to protect the branch artery, the use of “stilted building” embolization can achieve good therapeutic effects, and the short-term follow-up results are satisfactory; the technique can effectively protect branch arteries originating from aneurysms.

## 1. Introduction

Endovascular therapy is one of the main treatment methods for intracranial aneurysms. Coil embolization is widely used for ruptured and unruptured aneurysms and is capable of relatively high efficacy and safety [1–3]. Many aneurysms often have branch arteries, [4] and it is especially important to protect them during embolization. However, the protection of these arteries during aneurysm embolization is difficult [4–6] when the branch artery originates from the neck or the sidewall of the aneurysm and cannot be effectively protected by the balloon or stent. From June 2010 to June 2020, Beijing Chaoyang Hospital and Beijing Tiantan Hospital Affiliated with Capital Medical University received 25 patients with intracranial aneurysms involving branch arteries that were treated by embolization using the “stilted building” technique. The curative effect was satisfactory. The stilted buildings of the Chinese Tujia people feature a bamboo or wooden structure suspended above the ground and raised on timber stilts. The principle of the embolization technology is similar to that of stilted buildings, so it is named the “stilted building” technique and which was partial embolization [7]. A retrospective report and our technical experience are described below.

## 2. Materials and Methods

This is a retrospective study. From June 2010 to June 2020, a total of 25 patients with intracranial aneurysms with branch arteries were treated by coil embolization with the “stilted building” technique. The patient cohort included 7 males and 18 females aged 35 to 84 years, with a mean age of 63.5 ± 11.1 years. Eighteen patients had a spontaneous subarachnoid hemorrhage, nine with Hunt & Hess grade I, four with grade II, and five with grade III; the remaining 7 patients had no obvious symptoms, and their aneurysms were found during physical examination. The diagnosis was confirmed by plain head CT scan and digital subtraction angiography (DSA). Twelve patients were embolized with the “stilted building” technique alone, 11 with the “stilted building” technique combined with stent-assisted embolization, and 2 with the “stilted building” technique combined with balloon-assisted embolization.

### 2.1. Technical Details of the “Stilted Building” Technique

**Figure 1:**
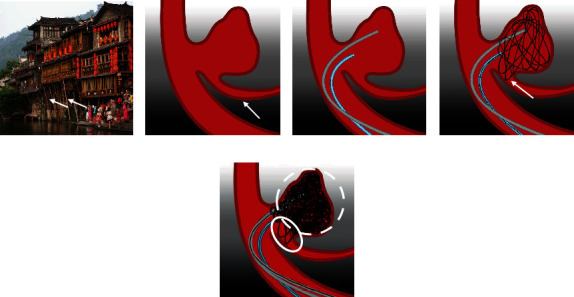
Technical details of the “stilted building” technique. (a) The stilted buildings of the Chinese Tujia people feature a bamboo or wooden structure suspended above the ground and raised on timber stilts (arrow). (b) A branch originates from the neck of the aneurysm (arrow), and its blood flow direction is at an acute angle with the parent artery. It is difficult to protect it with a balloon or stent during the embolization of the aneurysm. (c) The aneurysm is embolized by using the “stilted building” technique; first, a double microcatheter is placed in the appropriate part of the aneurysm. (d) A coil with a diameter slightly larger than the aneurysm is used to form a support frame within the aneurysm, allowing several rings to enter the branch artery to play a supporting role (arrow). (e) The ruptured sac and body continue to be embolized with the supporting frame, avoiding the branch artery. The presence of the supporting frame allows subsequent coils to be held in a suitable position without entering the branch artery. The pillars of the “stilted building” are indicated by the solid ellipse, and the body of the “stilted building” is shown by the dotted ellipse.

All aneurysm embolization procedures were performed by two interventional neurosurgeons and one interventional neuroradiologist. For patients with ruptured aneurysms undergoing emergency surgery, if stent-assisted embolization was required, tirofiban was administered by intravenous infusion before the stent was released. The initial dose was 10 *μ*g/kg, which was completely delivered within 3 minutes. Then, tirofiban was infused intravenously at a rate of 0.15 *μ*g/kg/min. After the operation, tirofiban was instilled at a maintenance dose for 24 hours. Patients with indwelling stents were routinely given aspirin 100 mg/day and clopidogrel 75 mg/day after surgery for 3 months. Patients undergoing elective surgery were prescribed aspirin 100 mg/day and clopidogrel 75 mg/day at least 5 days before their operation. After the operation, they continued to receive dual antiplatelet therapy (aspirin 100 mg/day and clopidogrel 75 mg/day) for 3 months and long-term administration of aspirin after 3 months. If a stent was not used, the patients were given aspirin 100 mg/day for 1 month. All treatments were performed under general anesthesia and systemic heparinization, with an activated clotting time of approximately 250-300 seconds. An arterial sheath was inserted into the femoral artery using Seldinger's technique, and a 6F or 8F guiding catheter (Envoy, Codman Neurovascular, USA) was positioned into the extracranial segment of the internal carotid artery. A microcatheter, such as a Headway 17 (MicroVention-Terumo, Tustin, CA, USA) or an Echelon 10 (Medtronic, Minneapolis, MN, USA), was placed in an appropriate position relative to the aneurysm through the guiding catheter. The aneurysm was embolized by using the “stilted building” technique alone or after placement of a Hyperglide occluded balloon catheter (Medtronic) or Rebar stent catheter (Medtronic) into the target artery as required. The principle of this technique is similar to that of the Tujia stilted buildings supported by timber stilts (Figure 1(a)). First, a larger diameter coil that is slightly larger than the diameter of the aneurysm was chosen to form a stable internal frame to support the sidewall of the aneurysm and the wall of the branch artery, resembling the pillars of the “stilted building” and providing a strong support point for the aneurysm to later be filled with coils (Figure 1(d)). After completing the preliminary frame which still contained substantial coil-free dead space due to the use of large-diameter coils, leaving much of the aneurysmal space unembolized, the aneurysm was then filled with additional smaller coils (Figure 1(e)). By changing the position of the microcatheter tube head and selecting coils of different properties and diameters, the distribution of the coils can be adjusted to form the building body of the “stilted building”, avoiding the area where the branch artery originates from the aneurysm (Figure 1). In addition, according to the type of aneurysm neck, an appropriate balloon or stent can be selected to form a more stable frame structure or to protect other branches.

### 2.2. Angiographic Evaluation

The location of the aneurysm was defined at the origin of the neck. The angiographic outcome was evaluated according to the Raymond classification [8, 9] immediately after the operation and at follow-up. Angiographic recurrence was defined as any worsening of the Raymond classification or enlargement of the residual aneurysm. By comparing the follow-up and intraoperative blood flow in the relevant arteries, the protective effects on the branches were evaluated. The follow-up period was defined as the number of postoperative months to recurrence or the latest angiogram. When performing DSA during the operation and the postoperative follow-up, a PHILIPS Allura Xper FD20 (Philips Healthcare, Best, the Netherlands) was used. The size of the aneurysm was measured with reconstructed 3D images by using an Allura 3D-RA workstation (Philips Healthcare, Best, the Netherlands).

### 2.3. Clinical Assessment

Clinical outpatient follow-up was performed at approximately six months and annually after treatment, and each patient was evaluated by the modified Rankin Scale (mRS) [10]. If possible, follow-up imaging with DSA, MRA, or CTA was performed. Perioperative complications were defined as any new neurologic deficits, serious adverse events extending hospitalization, or death occurring within 30 days after the operation.

## 3. Results

All 25 patients successfully underwent aneurysm embolization (Table 1). Of these, 9 aneurysms were located on the posterior communicating segment of the internal carotid artery (ICA), 11 aneurysms were located on the middle cerebral artery (MCA), 4 aneurysms were located on the anterior communication artery (AcomA), and 1 aneurysm was located on the basilar artery (BA). Furthermore, 12 aneurysms were located on the sidewall of the parent artery, and 13 were located at the artery bifurcation. During the operation, the ruptured sac and most of the body of the aneurysm were embolized using the “stilted building” technique. Immediate imaging showed that the blood flow to the branch arteries from the neck or sidewall of the aneurysm was unobstructed. The mRS scores of the 25 patients during the follow-up period were mRS 0 for twenty-one patients, mRS 1 for three patients, and mRS 6 for one patient. The DSA follow-up time was 0.5-12 months, with an average of 6.4 ± 3.0 months, and the follow-up rate was 36%. No aneurysms recurred among the patients who completed the follow-up. The branch arteries of 24 patients had normal blood flow and demonstrated no significant changes, while 1 patient showed related arterial blood flow restriction. Three patients (12%) were considered to have perioperative complications, all of which were related to ischemic perforator arteries. One patient, with an aneurysm at the bifurcation of the right MCA, developed right-sided facial paralysis and left limb weakness two weeks after surgery. MRI-DWI showed fresh infarction of the hind limb of the right internal capsule, no clear arterial occlusion, and blood flow restriction on DSA review. A second patient, with a left posterior communicating aneurysm, showed decreased right limb muscle strength and reduced consciousness on the first postoperative day. Emergency reexamination of the brain CT showed no significant increase in hemorrhage or new infarcts. The patient's state gradually recovered after enhanced fluid perfusion. Considering possible small embolus shedding and vasospasm, the patient's consciousness was clear at the time of discharge, and the right limb muscle strength was grade 4. The last patient, with a left posterior communicating aneurysm, developed blurred vision and hemianopia on the right side but had normal limb movement 2 weeks after surgery. Reexamination of the brain CT showed left occipital lobe infarction, and DSA showed that the blood flow of the left posterior communicating artery was restricted. Additionally, two patients had nonsurgery-related complications. One patient, with an aneurysm of the sidewall of the M1 segment of the right MCA, developed communicating hydrocephalus 12 months after surgery and underwent a lumbar cisternal-abdominal shunting. The second patient, with a right posterior communicating aneurysm, died of postoperative ventricular fibrillation.

### 3.1. Illustrative Cases

#### 3.1.1. Patient 1

**Figure 2:**
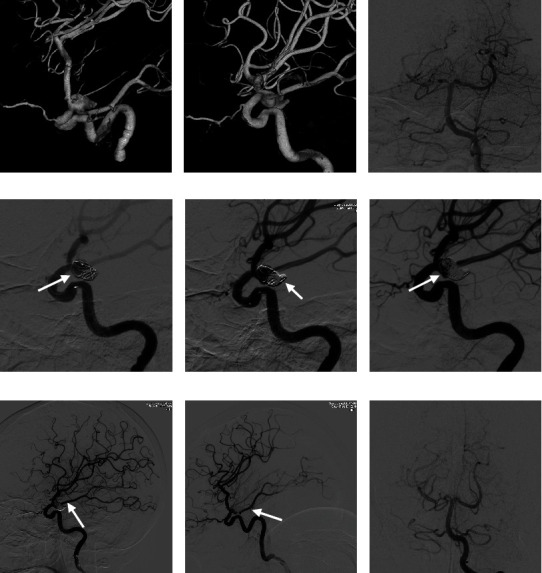
Cerebral angiography of the patient 1. (a–c) DSA examination revealed that a right posterior communicating ruptured aneurysm and the embryonic posterior cerebral artery originated from the neck of the aneurysm. (d) The first coil forms a stable support frame, and a few rings entered the posterior communicating artery to provide support (the pillars of the “stilted building”) (arrow). (e) The aneurysm continues to be embolized, and angiography shows that the ruptured sac is densely embolized (arrow). (f) To embolize the aneurysm body, the detaining effect of the frame was leveraged to adjust the position of the coils and avoiding the branch artery (body of the “stilted building”) (arrow). (g) Immediate angiography shows that the ruptured sac and body of the aneurysm are embolized. The lumen of the communicating artery is unobstructed (arrow). (h, i) Seven months after interventional surgery, follow-up DSA shows no recurrence of the aneurysm, and the lumen of the posterior communicating artery is unobstructed (arrow).

A 66-year-old woman presented with a symptomatic aneurysm of the posterior communicating segment of the right ICA. Head CT showed subarachnoid and intraventricular hemorrhage. DSA examination revealed that a right posterior communicating ruptured aneurysm measuring approximately 10 mm × 7 mm, with a neck width measuring approximately 7 mm, and the embryonic posterior cerebral artery originated from the neck of the aneurysm (Figures 2(a)–2(c)). The patient underwent intracranial aneurysm embolization under general anesthesia. First, assisted by a supersmooth guidewire, a 6F guiding catheter was indwelled into the extracranial segment of the ICA; the Headway 17 microcatheter and the Echelon 10 microcatheter were placed within the aneurysm with assistance from the microguidewire through the guiding catheter. The microguidewire was withdrawn. Then, the first coil, with a slightly larger diameter (9 mm × 24 cm) than the aneurysm, was fed through the Headway 17 microcatheter. A few rings were inserted into the posterior communicating artery to form a stable support frame (Figure 2(d)). Angiography showed that the lumen of the ICA and the posterior communicating artery was unobstructed. The coil was then released. Finally, a 4 mm × 10 cm MicroPlex Coil System (MicroVention-Terumo, Tustin, CA, USA) and 4 mm × 12 cm and 4 mm × 10 cm Jasper coils (Peijia Medical, Suzhou, Jiangsu, China) were continuously fed through the Echelon 10 microcatheter. Angiography showed that the ruptured sac was embolized (Figure 2(e)). The aneurysm continued to be filled with 6 mm × 15 cm, 4 mm × 6 cm, 4 mm × 6 cm, 5 mm × 15 cm, and 4 mm × 10 cm Jasper coils (Peijia Medical) through the Headway 17 microcatheter (Figure 2(f)). Immediate angiography showed that the ruptured sac of the aneurysm and the aneurysm body were embolized. The lumen of the communicating artery was unobstructed (Figure 2(g)). Head CT was reviewed after the operation, and no new bleeding was seen. Seven months after discharge, the mRS score was 0. Follow-up DSA showed no recurrence of the aneurysm, and the lumen of the posterior communicating artery was unobstructed (Figures 2(h) and 2(i)).

#### 3.1.2. Patient 2

**Figure 3:**
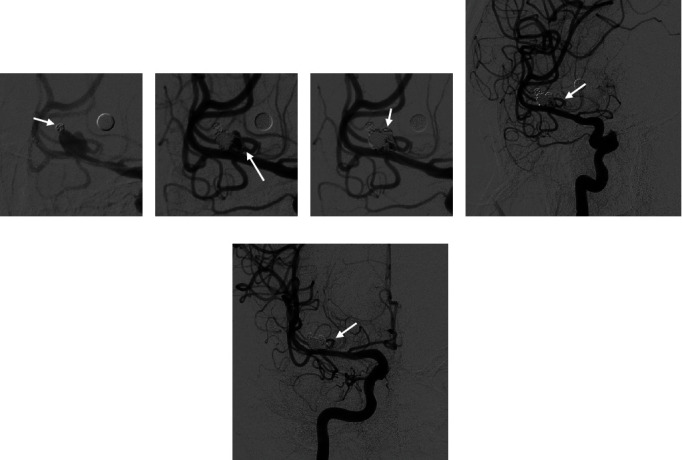
Cerebral angiography of the patient 2. (a) Embolization of the ruptured sac (arrow). (b) A few rings enter the branch artery to form stable support (pillars of the “stilted building”) (arrow). (c) The aneurysm continues to be filled with the coil, avoiding the branch from the body of the aneurysm (body of the “stilted building”) (arrow). (d) Immediate angiography shows that the ruptured sac and body of the aneurysm are embolized (Class IIIa [9]), and the lumen of the branch artery from the body of the aneurysm is unobstructed (arrow). (e) Five months after interventional surgery, follow-up DSA shows that the aneurysm had not recurred, and the branch lumen from the body of the aneurysm is unobstructed (arrow).

A 55-year-old man presented with a symptomatic aneurysm of the M1 segment of the right MCA. Head CT showed subarachnoid hemorrhage. DSA examination revealed a ruptured aneurysm of the M1 segment of the right MCA measuring 7.5 mm × 5.4 mm, with a neck width measuring approximately 5.7 mm, branch arteries originating from the body of the aneurysm supplying blood to the cortex, and the ruptured sac located at the bottom of the aneurysm. The other branch artery originated from the opposite wall of the parent artery. First, after placing the stent microcatheter and embolization microcatheter, multiple smaller coils were inserted through the Headway 17 catheter to embolize the ruptured sac (Figure 3(a)), and then, the distal half of the aneurysm was embolized. Then, a larger coil was selected to embolize approximately all of the remaining half to form a frame to support the sidewall of the aneurysm, and a few rings were entered into the branch artery from the aneurysm body to form the stable support (Figure 3(b), arrow). A 4 × 20 mm Solitaire stent (Medtronic) was placed to protect the neck and the large branch arteries from the opposite wall of the parent artery. With the support of the frame formed by the larger coil, the aneurysm continued to be filled with the coil, avoiding the branch from the body of the aneurysm (Figure 3(c)). Immediate DSA showed that the ruptured sac and body of the aneurysm were embolized, and the lumen of the branch artery from the body of the aneurysm was unobstructed (Figure 3(d)). The patient was reexamined 5 months after the operation. The mRS score was 0. Follow-up DSA showed that the aneurysm had not recurred, and the branch lumen from the sidewall of the aneurysm was unobstructed (Figure 3(e)). The patient had no complications.

## 4. Discussion

Endovascular aneurysm treatment includes coil embolization, balloon-assisted coil embolization, stent-assisted coil embolization, flow diverters, and intrasaccular flow disrupters. [11–14] In recent years, the technology for performing endovascular treatment of intracranial aneurysms has made great progress. The wider application of occlusion balloon- and stent-assisted embolization technology has greatly improved success in protecting branching arteries when embolizing aneurysms [15–19]. When the branch artery originates from the sidewall of the aneurysm and is very close to the aneurysm neck, an occlusion balloon or stent can protect the branch artery. However, when the angle between the branch artery of the aneurysm and the parent artery is less than 90° or when the diameter of the branch artery is small (<1 mm), it is difficult to release a stent and to ensure that the stent is completely inserted and fully open. In these cases, the use of the “stilted building” technique is very effective.

In the “stilted building” technique, the first set of larger coils form a frame in the aneurysm cavity, and one or two rings from the coils protrude into the branch artery to protect it and act as a support structure. The first set of coils can refer to the first coil alone or a group of coils; for aneurysms with necks that are too wide or extremely irregular in shape, the formation of a stable and uniform frame may require multiple coils with the help of multimicrocatheter technology. The main purpose of the “stilted building” technique is to palliatively or radically embolize the aneurysm while protecting the branch arteries adjacent to the aneurysm. The first set of slightly larger coils supports the wall of the branch artery and the wall of the aneurysm to stabilize the overall structure and form the pillars of the “stilted building.” The subsequent coils focus on embolizing any ruptured sacs and the aneurysm body while avoiding the branch arteries, that is, the “body” of the “stilted building.” The main points of the “stilted building” technique are as follows: First, the formation of a support frame, that is, from the frame attached to the wall of the aneurysm cavity, one or two rings from the coils (the pillars of the “stilted building”) are allowed to enter the branch artery to protect it and provide support. The support point is located at the bifurcation of the artery at the neck of the aneurysm or within the branch itself. The idea of “support” means that the rings of the coil protruding into the branch artery should have a sufficient area to adhere to the artery wall rather than float within the artery cavity; then, given the support and restrictiveness of the frame coil, subsequent coils can be held within the aneurysm cavity (the body of the “stilted building”) and are in no danger of entering into the supported artery. In most cases, the “stilted building” technique requires a combination of dual microcatheter, balloon, or stent assistive technology. When using dual microcatheter technology, one microcatheter is mainly used to form the supporting frame, and the other is mainly used to fill the aneurysm cavity. Balloons or stents are generally used to protect the main trunk or a branch of the parent artery, and an additional branch is protected by the “stilted building” technique.

A total of 12 patients in this group had aneurysms located on the sidewall of the parent artery. It was difficult for the microcatheter to enter the branch artery due to the angle. Furthermore, the diameter of some of the branch arteries was very small, making it difficult to release the stent and for balloons to protect the branch arteries. In this way, the first set of coils, which were relatively larger than the diameter of the aneurysm, were leveraged to support the branch artery wall and the aneurysm wall to form a frame, and the distribution of the subsequent coils was then adjusted to continue embolizing the aneurysm body. In some cases, the aneurysm embolization procedures were Class III according to the Raymond classification [8, 9]. However, since the residual aneurysm has a branch artery, a shunting effect for the blood flow was generated that could reduce the impact of the pressure of the blood flow on the remaining wall of the aneurysm, reducing the possibility of aneurysm recurrence in the residual. There does remain a possibility of recurrence; however, so closer angiographic follow-up is required. The short-term follow-up results in this group were satisfactory, but the DSA follow-up rate was relatively low. In the remaining 13 patients in this group, the aneurysm was located at the bifurcation of the parent artery, and most branch arteries originated from the neck of the aneurysm. There are many treatment methods for this type of aneurysm, including clipping, stent-assisted embolization, and balloon protection embolization; if the diameter of the artery lumen is sufficiently large, the branch artery can also be protected by inserting a single stent or a “Y” stent [20–22]. However, because these patients were older and in the acute stage of aneurysm rupture, we chose the “stilted building” technique to embolize the aneurysm. DSA follow-up showed that the embolization structure was very stable, and the branch arteries involved in the aneurysm had good patency.

When using the “stilted building” technique, a small part of the coils will be distributed near the beginning of the branch artery, which may affect the blood flow to that artery. However, the coverage of the metal coils is theoretically lower than that of the stent. The probability of the stent affecting the blood flow of the branch arteries that it covers is very low, and even LVIS stents, with their greater metal coverage, are relatively safe [23, 24]. Therefore, we can infer that the “stilted building” technique has only a limited effect on the blood flow of the branch artery. Additionally, the DSA follow-up results in this group also confirmed that the coil structure was stable and that the blood flow of the branch arteries did not change significantly. Furthermore, if the aneurysm was wide necked, the “stilted building” technique had to be combined with stent-assisted embolization. Considering that the stent may affect the blood flow of the branch arteries, we used the Solitaire stent with a larger mesh; even if the blood flow to the branch arteries is affected later, it would buy time for vascular compensation. Eleven patients underwent stent-assisted embolization for their aneurysms using the “stilted building” technique. Among the patients followed up by DSA, these aneurysms did not recur. When using this technique, thromboembolic complications may occur. Routine heparinization during surgery and the use of dual antiplatelet drugs after surgery reduced the risk of thrombosis. Because the ruptured sac of the aneurysm and most of the aneurysm body were embolized, the probability of rebleeding from the aneurysm was theoretically very low. In this group of 25 patients, no rebleeding occurred in the short-term outpatient follow-up after the operation. However, the sample size was small, and the follow-up rate for DSA was relatively low. Further follow-up is needed to assess the long-term efficacy.

## 5. Conclusions

In an aneurysm with a branch artery, when a balloon or stent cannot be effectively used to protect the branch artery, the use of “stilted building” embolization can achieve good therapeutic effects, and the short-term follow-up results are satisfactory; the technique can effectively protect branch arteries originating from aneurysms.

## Figures and Tables

**Table 1:** Patient, aneurysm, procedure, and follow-up data.

	Stilted building technique	Stilted building technique +SAC	Stilted building technique +BAC	Total
*General*				
Sex				
Men	3	3	1	7
Women	9	8	1	18
Age (years)	66.2 ± 10.5	62.2 ± 8.2	55 ± 20	64 ± 11
*Aneurysm characteristics*				
Aneurysm location				
AcomA	4	0	0	4
MCA	3	7	1	11
PcomA	5	3	1	9
BA	0	1	0	1
Relationship with the parent artery				
Sidewall	6	5	1	12
Bifurcation	6	6	1	13
Aneurysm size (mm)	5.4 × 4.9	6.9 × 5.9	10.1 × 8.8	6.6 × 5.8
Rupture status				
Ruptured	9	7	2	18
Unruptured	3	4	0	7
Branch/origin				
A2/aneurysm neck	4	0	0	4
/aneurysm sidewall	0	0	0	0
M2/aneurysm neck	2	5	1	8
/aneurysm sidewall	0	2	0	2
PcomA/aneurysm neck	2	2	1	5
/aneurysm sidewall	3	1	0	4
Others				
Subsidiary MCA	1			1
Pericallosal artery	1			1
PCA, SCA		1		1
Central sulcus artery		1		1
*Follow-up*				
Perioperative complications				
Hemorrhage	0	0	0	0
Ischemia	1	2	0	3
Others	0	0	0	0
DSA				
Follow-up rate	8%	64%	100%	36%
Average time (month)	7	7.1	6.5	6.4
Outcome	One patient had limited blood flow in the left posterior communicating artery; the remaining patients had no recurrence and the branches were unobstructed	No recurrence and the branches were unobstructed	No recurrence and the branches were unobstructed	
mRS				
0	11	8	2	21
1	1	2	0	3
2	0	0	0	0
3	0	0	0	0
4	0	0	0	0
5	0	0	0	0
6	0	1(death from ventricular fibrillation)	0	1
Total	12	11	2	25

^∗^AcomA: anterior communication artery; MCA: middle cerebral artery; PcomA: posterior communication artery; BA: basilar artery; PCA: posterior cerebral artery; SCA: superior cerebellar artery.

## Data Availability

The datasets used and/or analyzed during the current study are available from the corresponding author on reasonable request.
